# Prediction of Microvascular Invasion in Hepatocellular Carcinoma: Preoperative Gd-EOB-DTPA-Dynamic Enhanced MRI and Histopathological Correlation

**DOI:** 10.1155/2018/9674565

**Published:** 2018-01-23

**Authors:** Mengqi Huang, Bing Liao, Ping Xu, Huasong Cai, Kun Huang, Zhi Dong, Ling Xu, Zhenpeng Peng, Yanji Luo, Keguo Zheng, Baogang Peng, Zi-Ping Li, Shi-Ting Feng

**Affiliations:** ^1^Department of Radiology, The First Affiliated Hospital, Sun Yat-Sen University, 58 Second Zhongshan Road, Guangzhou, Guangdong 510080, China; ^2^Department of Pathology, The First Affiliated Hospital, Sun Yat-Sen University, 58 Second Zhongshan Road, Guangzhou, Guangdong 510080, China; ^3^Faculty of Medicine and Dentistry, University of Western Australia, Perth, WA, Australia; ^4^Department of Hepatobiliary Surgery, The First Affiliated Hospital, Sun Yat-Sen University, 58 Second Zhongshan Road, Guangzhou, Guangdong 510080, China

## Abstract

**Objective:**

To investigate the imaging features observed in preoperative Gd-EOB-DTPA-dynamic enhanced MRI and correlated with the presence of microvascular invasion (MVI) in hepatocellular carcinoma (HCC) patients.

**Methods:**

66 HCCs in 60 patients with preoperative Gd-EOB-DTPA-dynamic enhanced MRI were retrospectively analyzed. Features including tumor size, signal homogeneity, tumor capsule, tumor margin, peritumor enhancement during mid-arterial phase, peritumor hypointensity during hepatobiliary phase, signal intensity ratio on DWI and apparent diffusion coefficients (ADC), T1 relaxation times, and the reduction rate between pre- and postcontrast enhancement images were assessed. Correlation between these features and histopathological presence of MVI was analyzed to establish a prediction model.

**Results:**

Histopathology confirmed that MVI were observed in 17 of 66 HCCs. Univariate analysis showed tumor size (*p* = 0.003), margin (*p* = 0.013), peritumor enhancement (*p* = 0.001), and hypointensity during hepatobiliary phase (*p* = 0.004) were associated with MVI. A multiple logistic regression model was established, which showed tumor size, margin, and peritumor enhancement were combined predictors for the presence of MVI (*α* = 0.1). *R*^2^ of this prediction model was 0.353, and the sensitivity and specificity were 52.9% and 93.0%, respectively.

**Conclusion:**

Large tumor size, irregular tumor margin, and peritumor enhancement in preoperative Gd-EOB-DTPA-dynamic enhanced MRI can predict the presence of MVI in HCC.

## 1. Introduction

 Hepatocellular carcinoma (HCC) is the 3rd leading cause of cancer related death worldwide and it is most frequently reported in East Asia [1]. HCC frequently presents during its advanced stages and the survival rates remain poor with a 5-year recurrence rate of 25% after liver transplantation and 70% after hepatic resection [2–4]. The size, number, and nuclear grade of the tumors, presence of vascular invasion, and severity of liver disease are all regarded as important predictors for recurrence and hence poor survival outcomes [5, 6]. Vascular invasion is considered as an important prognostic factor for tumor metastasis [7]. HCC has a propensity to spread intrahepatically via macroscopic or microscopic tumor emboli [8]. The risk of recurrence after liver transplant was 4.4-fold higher in patients with presence of microvascular invasion (MVI) and 15-fold higher in patients with presence of macrovascular invasion [9]. Macrovascular invasion can be detected on imaging such as contrast enhanced CT or MRI [7]. In comparison, MVI can be difficult to detect in the preoperative imaging such as conventional CT or MRI; hence histological confirmation after surgical resection is important [10]. An accurate preoperative estimation of MVI presence can help surgeons to choose appropriate surgical procedures for patients based on their risk-benefit assessment [11]. If liver resection is considered for patients with a high risk of MVI, a larger margin might be the preferred during curative resection [12]. For liver transplantation in patients with HCC, the absence of MVI has been proven to be an essential variable to predict better survival outcomes according to the new inclusion criteria [13]. Furthermore, patients who were predicted to have MVI are suitable candidates for studies that evaluate the effectiveness of neoadjuvant therapy [11]. Thus, accurate preoperative detection of MVI in HCC is important.

Unlike conventional enhanced MR, Gd-EOB-DTPA is a liver-specific intracellular MRI contrast agent that is initially distributed into the vascular and extracellular spaces [14] and provides a special hepatocellular parenchymal contrast during late-phase images. This unique property was found to improve sensitivity and specificity in the detection and classification of liver lesions compared to CT images and conventional MR images [15]. Thus, Gd-EOB-DTPA enhanced MRI can provide more valuable information for the assessment of HCC and has been widely used in preoperative evaluation settings.

Tumor size and imaging features, such as irregular tumor margins, peritumor enhancement and peritumor hypointensity during hepatobiliary phase, DWI, and apparent diffusion coefficients (ADC) values [4, 7, 16–20] have been previously suggested as predictors of MVI. Although certain imaging features in CT or nonspecific enhanced MR, such as larger tumor size, irregular tumor margins, and peritumor enhancement, may also suggest correlation with MVI [4], these features may vary in hepatic specific contrast MRI. Currently, there is no reported research which demonstrates a predictive model which will combine these imaging features, together with the unique functional and quantitative features using Gd-EOB-DTPA-dynamic enhanced MRI.

In this study, we aim to investigate whether the imaging features identified during the preoperative Gd-EOB-DTPA-dynamic enhanced MRI can predict the presence of MVI in patients with HCC. By using the data obtained, we hope to build a prediction model for future clinical use.

## 2. Materials and Methods

### 2.1. Patients

This retrospect study was approved by the institutional review board, and the requirement for informed consent was waived. The radiological, surgical, and histological database was reviewed from December 2011 to June 2015.

Patients were selected for this study based on the following inclusion criteria: (1) preoperative Gd-EOB-DTPA-dynamic enhanced MR imaging was performed, (2) there is no prior surgical or medical treatment, (3) hepatic resection or transplantation was performed within 1 month after preoperative imaging, and (4) HCC was confirmed on histopathology.

A total of 60 patients with 66 HCC lesions were included (54 males and 6 females, mean age was 52.2 years, ranging from 28 to 84 years). In this study the presence or absence of MIV was confirmed on the histopathology. This is defined as the presence of tumor cells within the vascular space lined by endothelium that was visible only on microscopy [21].

### 2.2. Image Acquisition

MRI examinations were performed using a 3.0 T MR system (Magnetom Verio, Siemens Healthcare, Erlangen, Germany), body coil, and supine position. Gd-EOB-DTPA-dynamic enhanced MR scanning protocol included the following sequences: the conventional T2WI, T1WI, and fat-suppressed T1WI; the dynamic enhanced images were 3 arterial phases, 3 portal venous phases, 1 delayed phase, and 20 min hepatobiliary phase which were scanned in fat-suppressed VIBE T1WI. Part of these patients' scanning protocol has included some additional sequences, such as DWI and T1 mapping (Table 1).

DWI was performed 10 min after Gd-EOB-DTPA was injected with a single-shot spin-echo planar sequence using tridirectional motion-probing gradients with 3 *b*-values of 50, 400, and 800 s/mm^2^. The parameters were listed as follows: TR/TE of 5300/83 ms; slice thickness of 5.5 mm; matrix size of 192 × 192; FOV of 380 × 380 mm; bandwidth of 1736 HZ/Px; scanning time of 90 s.

For acquisition of T1 mapping images, a dual flip-angle 3D gradient-echo sequence with volumetric interpolated breath-hold examination (VIBE) was performed before and 20 minutes after injection of Gd- EOB-DTPA. The parameters were listed as follows: TR/TE of 4.4/1.2 ms; slice thickness of 2 mm; flip angle of 2° and 11°; matrix size of 154 × 256; FOV of 248 × 330 mm. A parallel imaging technique (*R* = 2) was performed using generalized auto calibrating partially parallel acquisition.

T1 relaxation time estimates can be obtained with a simple algebraic expression:

(1) $$\begin{array}{c} T1=2TR\frac{S1/\alpha 1-S2/\alpha 2}{S2\alpha 2-S1\alpha 1}. \end{array}$$

(T1 means the T1 relaxation time of tissue; *S*1 and *S*2 were two VIBE signals at two different flip angles of *α*_1_ and *α*_2_; TR is repetition time.)

### 2.3. Image Analysis

All images were assessed by 2 abdominal radiologists with 2 and 15 years of experience in abdominal radiology. Both radiologists were blinded to the results of the histopathological findings. In patients with more than 1 HCC lesion, imaging findings of each lesion were assessed independently in accordance with the histopathology reports of each corresponding lesion.

2 trained radiologists reviewed all MR images and evaluated tumor size, signal homogeneity in T2WI sequence, tumor capsule, tumor margin, peritumor enhancement during mid-arterial phase, and peritumor hypointensity during hepatobiliary phase independently. Signal intensity ratio on DWI and ADCs, T1 relaxation times, and the reduction rate between pre- and postcontrast enhancement images were calculated by 1 trained radiologist.

The presence of tumor capsule was assessed during the equilibrium phase by identifying a thin, linear, enhanced structure surrounding the tumor. This was categorized into 3 groups depending on radiological appearance: completely surrounded tumor capsule, incompletely surrounded tumor capsule, or absence of tumor capsule.

Tumor margins were analyzed during both axial and coronal hepatobiliary phases and were grouped into three categories: smooth margin, which presents as a smooth tumor-normal liver interface; focal outgrowth, which presents as 2 or less nodules protruding into the normal liver parenchyma; multifocal outgrowths protruding into the normal liver parenchyma; fuzzy margin, which presents as patchy-like growth lesion with unclear margin.

Peritumor enhancement was defined as a patchy or crescent peritumor hyperintense region during the mid-arterial phase and isointense one during the portal or delayed phase [16].

Peritumor hypointensity was defined as an irregular, wedge-shaped, or flamed-like hypointense area of liver parenchyma outside the tumor margin during hepatobiliary phase. For majority of cases, this was seen as an area of relative hypointensity compared to the surrounding parenchyma. In minority of hyperintense HCC cases, this was seen as less hyperintense area when compared to the tumor itself or the presence of a hypointense rim (tumor capsule) [7].

**Figure 1:**
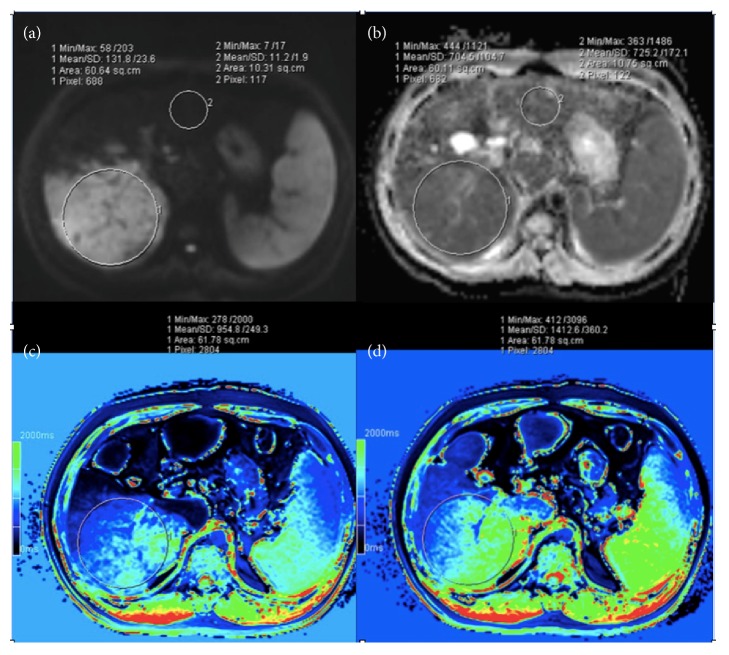
(a–d) Examples of placement of regions of interest (ROIs) in the same area of HCC lesion (ROI 1) and liver parenchyma (ROI 2) on a high-*b*-value DWI (a) and corresponding ADC maps (b) and ROIs of HCC lesion on T1 map before (c) and after (d) Gd-EOB-DTPA injection.

For quantitative analysis of DWI (with *b*-value of 800 s/mm^2^), regions of interest (ROIs) were drawn in the largest slice of HCCs and hepatic parenchyma at the workstation. The area of ROI in each HCC was set to include the entire lesion, not excluding components with different intense values [20]. The ROI in each liver was placed in the normal liver parenchyma at the same level of the HCC lesion while avoiding hepatic blood vessels. The lesion-to-liver SI (signal intensity) ratio on DWI was calculated using following equation: SI ratio = SI lesion/SI liver. ADC values were measured through the placement of ROIs in the HCCs and hepatic parenchyma on ADC maps. To ensure the identical placement of ROIs on DWI and ADC maps, ROIs were carefully copied from DWI and positioned at the same regions on the corresponding ADC maps (Figure 1).

T1 mapping was generated in postprocessing workstation (SyngoMMWP VE36A, Siemens). The ROI was drawn in the largest slice of the HCC lesion and contained the whole lesion in T1 mapping images (Figure 1). T1 relaxation time was calculated before and after administration of the contrast medium, respectively. Meanwhile, the reduction rate of T1 values was calculated as the percentage of the difference of T1 value before and after contrast (hepatobiliary phase) compared to the T1 value before contrast (reduction rate of T1 values = (T1pre − T1post/T1pre) × 100%) [22].

### 2.4. Pathology Analysis

All the original histopathology samples were reviewed retrospectively by an experienced pathologist (with 20 years of experience in liver pathology) blinded to the imaging findings. MVI was defined as tumor cells within vascular space lined by endothelium which was visible only on microscopy [11].

### 2.5. Statistical Analysis

The interobserver differences between the two trained observers were evaluated using the kappa agreement test. The inconsistent evaluation between the two observers was confirmed by a third experienced radiologist. A univariate logistic regression analysis was used in every measured parameter in this study and to select the statistically significant parameters. Then, all the significant parameters were entered into a further multiple logistic regression analysis to build a prediction model of MVI. The sensitivity and specificity were calculated for the parameters that showed statistical significance by multivariate analysis. *p* < 0.05 was considered to indicate a statistically significant difference in the univariate analysis, and *p* < 0.1 was considered to indicate a statistically significant difference in the multivariate analysis in this preliminary study.

## 3. Results

A total of 66 HCC lesions were analyzed in 60 patients with preoperative Gd-EOB-DTPA-dynamic enhanced MR imaging. 10 patients were found having more than 1 lesion. Of these 10 patients, 6 patients had 2 pathologically proven HCC lesions and all the 12 lesions were subsequently analyzed. The other 4 patients have intrahepatic satellite lesions and only the 4 main lesions were analyzed. 6 patients with multiple HCC underwent liver transplantation. Various imaging features with or without presence of MVI were shown in detail in Table 2.

### 3.1. Accuracy Statistics between Observers

The interobserver agreement test has demonstrated that the imaging feature assessment of the tumor capsule and tumor margin reached a moderate agreement (0.454, 0.513, resp.), the assessment of the signal homogeneous and peritumor hypointensity reached a substantial agreement (0.683, 0.782, resp.), and the peritumor enhancement reached an almost perfect agreement (0.822) (Table 3) [23].

### 3.2. Indicators of MVI

**Figure 2:**
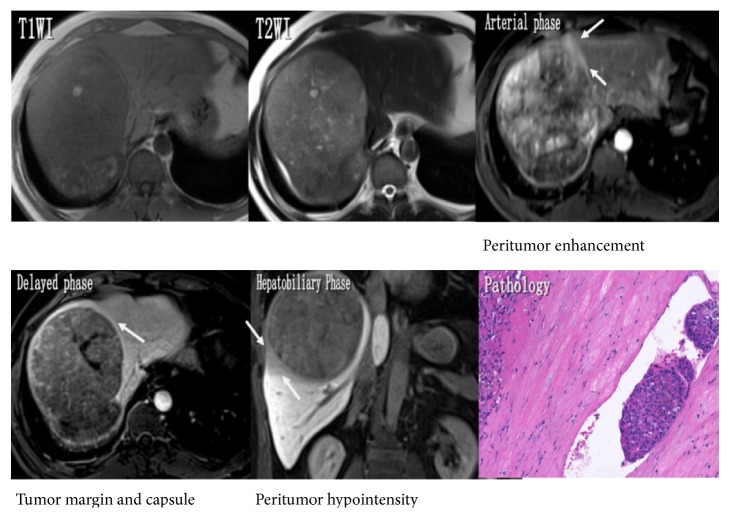
A 47-year-old man with pathologically proved MVI. The maximum diameter of the tumor in the right liver lobe was 143 mm, and the axial T2WI has shown heterogeneous signal. A patchy peritumor hyperintense region was shown in the mid-arterial phase and isointense one in the delayed phase. A not completely surrounded tumor capsule and focal outgrowth of nodules were shown in the delayed phase. A wedge-shaped hypointense area of liver parenchyma which located outside the tumor margin was seen in the coronal hepatobiliary phase image.

**Figure 3:**
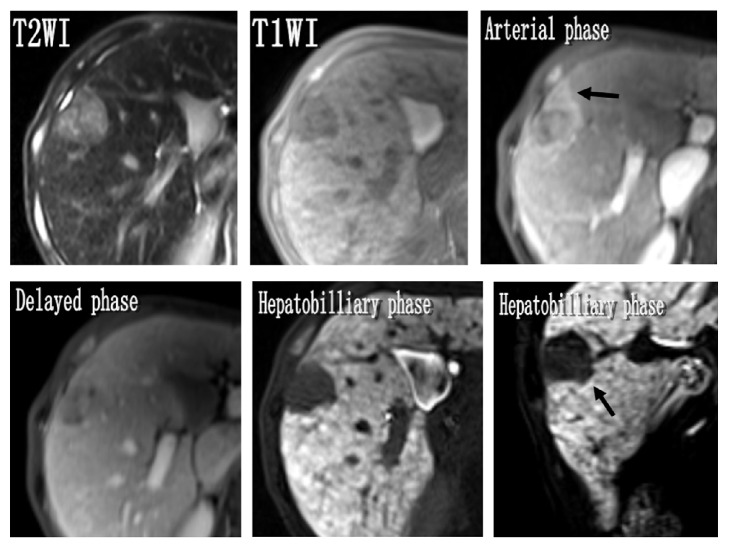
A 55-year-old man with pathologically proved MVI. The maximum diameter in the right liver lobe was 30 mm with a flam-like peritumor enhancement and not completely surrounded tumor capsule and focal outgrowth of nodules in the delayed phase but no peritumor hypointensity in hepatobiliary phase image.

The univariate logistic regression analysis of the 66 HCCs has shown that the maximum tumor diameter, tumor margin, peritumor enhancement, and peritumor hypointensity during hepatobiliary phase demonstrated statistically significant correlation with MVI (*p* < 0.05) (Figures 2 and 3). Tumor margin, peritumor enhancement, and peritumor hypointensity, in particular, demonstrated higher risk for the presence of MVI (OR: 4.247, 7.150, and 6.000, resp.). On the other hand, features such as the signal homogeneity during T2WI sequence, tumor capsule, DWI and ADCs, T1 relaxation times, and the reduction rate did not demonstrate significant correlation with the presence of MVI (*p* > 0.05) (Table 4).

Univariate logistic regression analysis of the different types of tumor margin showed that nonsmooth margin was the only statistically significant predictor for MVI in histopathology (*p* = 0.006); there was no statistical difference between the three different types of nonsmooth margin (*p* = 0.999).

Furthermore, the multiple logistic regression analysis of the significant parameters showed that a prediction model which consisted of larger tumor diameter, irregular tumor margin, and peritumor enhancement was significantly related to the presence of MVI (*α* = 0.1), with a sensitivity of 52.9% and specificity of 93.0%. *R*^2^ of this prediction model was 0.353. Moreover, the peritumor enhancement was the most important risk factor in this model, with an OR of 6.065 and 95% CI of 1.131 to 32.516 (Table 5).

## 4. Discussion

This study focused on the predictive value of MVI based on morphological and functional imaging features using preoperative Gd-EOB-DTPA-dynamic enhanced MRI. This was different from previous studies where imaging features were evaluated using CT or conventional MR imaging. In addition, the Gd-EOB-DTPA-dynamic enhanced MRI involves the use of a special hepatocellular parenchymal contrast during late-phase images which has been found in previous studies to improve the sensitivity and specificity in the detection and classification of liver lesions compared to other contrast CT images or conventional MR images alone [15]. Moreover, multiple logistic aggression analysis was performed during our study to take the interactions of those parameters into consideration instead of univariate analysis. Our interobserver agreement test has demonstrated moderate to almost perfect agreement during independent evaluation which indicates the reliability and applicability of these imaging features as reliable predictors for the presence of MVI.

In our study, 4 parameters (tumor size, tumor margin, peritumor enhancement, and peritumor hypointensity during hepatobiliary phase) have been proven to have statistically significant correlation with the presence of MVI through the univariate logistic analysis. Further multiple logistic analyses have demonstrated substantial correlation between 3 parameters with the presence of MVI, excluding peritumor hypointensity.

Larger tumor size was one of the predictors of MVI in HCC but not an indicator for high risk disease where OR of the tumor size in this study was approximately equal to 1.0. There are conflicting opinions regarding the usefulness of tumor size alone in predicting MVI in HCC [4]. The result in this study was consistent with some previous studies [4, 24]. However, some previous studies have reported that the tumor size was associated with MVI during the univariate analysis but not in multivariate analysis [7, 16]. The reason of this inconsistency may be selection bias in the size of HCC lesions.

Tumor margin was demonstrated to be an important factor in our study that is highly predictive of MVI, which was consistent with the findings of Renzulli et al. [4] and Chou et al. [16]. However, our study has shown that only irregular margin was a statistically significant predictor for the presence of MVI (*p* = 0.006) regardless of its subtypes (i.e., focal protruding nodules, multiple protruding nodules, or fuzzy margin). However, unlike Renzulli et al. [4] or Chou et al. assessments of HCC lesions were done using either CT or both CT and MR with nonspecific contrast agents [16], which demonstrated that the presence of focal infiltration was closely associated with MVI [4]. In our study, the features were analyzed during hepatobiliary phase and margin in this phase may be more clear than CT images, leading to an overassessment of the tumor margin, which means, for example, one lesion with one focal protruding in CT may be seen as one with multiple protruding during hepatobiliary phase in d-EOB-DTPA-dynamic enhanced MRI.

Peritumor enhancement was highly predictive of MVI, which was consistent with previous studies [4, 25]. Kim et al. suggested that an irregular circumferential peritumor enhancement might represent a direct tumor-related hemodynamic change in the corona enhancement and/or the tumorous AP shunt where irregular circumferential peritumor enhancement was the only statistically significant risk factor for MVI [25]. Consistent with previous studies, our study also suggests the presence of peritumor enhancement is likely due to the compensatory arterial hyperperfusion that occurred in the areas of decreased portal flow secondary to minute portal branch occlusion from tumor invasion [4]. Peritumor enhancement in our study was assessed during dynamic enhanced phases which included 3 arterial phases and 2 portal venous phases and 1 delayed phase where it can be more easily detected than general CT imaging [4].

Peritumor hypointensity during hepatobiliary phase was related to the presence of MVI when using univariate regression logistic analysis but not during the multiple regression analysis, while results from Kim et al. show that the peritumor hypointensity in EOB-MRI can be useful in predicting MVI preoperatively [7]. The relationship of peritumor hypointensity and MVI may be explained by the possible decreased uptake of GD-EOB-DTPA in hepatocytes supplied by minute portal branches which is obstructed by tumor thrombi resulting in hemodynamic changes [7]. Kim et al. concluded similar explanation between relationship of peritumor enhancement and MVI. The lack of relationship between peritumor hypointensity and MVI using multiple regression analysis may be explained by the independent occurrence of peritumor hypointensity and peritumor enhancement in our study. However, these 2 features did not always present at the same time; hence further research looking at the significance of peritumor hypointensity and peritumor enhancement is required to build a more accurate predictor model of MVI.

The signal homogeneity, tumor capsule, DWI and ADCs, T1 relaxation times, and the reduction rate were proven to have no significant relationship with MVI. Previous study has demonstrated that DWI findings can be useful for preoperative prediction of MVI and HCCs with MVI having shown higher SI ratios and lower ADC values than HCCs without MVI [26]. The difference may be due to the larger lesions in our research which represent high degree of degeneration and necrosis. At the same time, the small number of cases with DWI and ADC sequences may be another limitation factor. T1 relaxation time and the reduction rate were often used in assessment of liver function and cirrhosis [22, 27]. To our best knowledge, no previous studies have been done using T1 mapping to predict MVI of HCC. In our study, T1 relaxation time and the reduction rate were not found to correlate with the presence of MVI; whether these two factors would help to predict MVI is hard to assess due to the limited case number (only 27 cases).

We noted that our study has several limitations. Firstly, the possibility of a selection bias cannot be excluded due to its retrospective nature. Secondly, the study is limited by its small sample size of HCC lesions where the recommended sample number for multiple regression logistic analysis is about 20 times the number of parameters. Considering the above 2 limitations, it may result in an incomplete representation of all HCC radiological features. Thirdly, many parameters have been previously explored, but the correlation between these parameters, especially peritumor enhancement and peritumor hypointensity, has not been previously evaluated. Therefore, further research assessing these two factors is required to build a more accurate prediction model of MVI.

## 5. Conclusion

In conclusion, larger tumor size, irregular tumor margins, and the presence of peritumor enhancement and peritumor hypointensity during hepatobiliary phase can predict the presence of MVI independently. Furthermore, a prediction model combining tumor size, margin, and peritumor enhancement in preoperative Gd-EOB-DTPA dynamic enhanced MRI can predict the presence of MVI with low sensitivity but high specificity. Consequently, the radiological prediction of MVI before treatment is feasible. An accurate preoperative estimation of MVI presence can help determine more appropriate surgical strategies for patients based on more accurate risk-benefit assessments.

## Figures and Tables

**Table 1:** Sequences and parameters of Gd-EOB-DTPA-dynamic enhanced MRI.

Sequences	Image plane	TR/TE (msec)	FOV (mm)	Flip angle	Thickness (mm)	Matrix	Scanning order
T1WI	A^*∗*^	225/2.2	258 × 330	70	6	200 × 320	1
Dixon	A^*∗*^	5.5/2.5	380 × 380	65	3	320 × 320	2
T2WI	A^*∗*^	2000/91	350 × 350	150	6	410 × 512	3
T1-mapping	A^*∗*^	4.4/1.2	248 × 330	2,11	2	154 × 256	4
VIBE^*∗*^	A^*∗*^	3.3/1.2	248 × 330	13	2	96 × 256	5
DWI & ADC	A^*∗*^	5300/83	380 × 380	/	5.5	192 × 192	6
HBP	A^*∗*^	2000/78	380 × 380	13	5.5	380 × 380	7
HBP	C^*∗*^	1600/83	380 × 380	13	5	320 × 320	8
T1 mapping	A^*∗*^	4.4/1.2	248 × 330	2,11	2	154 × 256	9

^*∗*^A: axial; C: coronal; ^*∗*^VIBE: volumetric interpolated breath-hold examination, including nonenhanced imaging, 3 arterial phases, 3 portal venous phases, and 1 delayed phase; HBP: hepatobiliary phase.

**Table 2:** Radiologic imaging features of 66 HCCs and MVI.

Parameters	Negative	Positive
*Signal homogeneity*		
Homogenous	21 (0.43)	4 (0.23)
Heterogeneous	28 (0.57)	13 (0.77)
*Tumor capsule*		
Completely surrounded	18 (0.36)	2 (0.12)
Not completely surrounded	22 (0.45)	11 (0.64)
None	9 (0.18)	4 (0.24)
*Tumor margin*		
Smooth margin	11 (0.22)	0
Focal protruding	9 (0.18)	2 (0.12)
Multiple protruding	28 (0.57)	12 (0.70)
Fuzzy margin	1 (0.02)	3 (0.18)
*Peritumor enhancement*		
No	39 (0.79)	6 (0.35)
Yes	10 (0.21)	11 (0.65)
*Peritumor hypointensity*		
No	35 (0.71)	5 (0.29)
Yes	14 (0.29)	12 (0.71)
ADC (HCC, ×10^−3^ mm^2^/s)	1.071	0.995
*T*1pre (ms)	1111	1634
*T*1post (ms)	705	1047
ΔT1	0.41	0.37

**Table 3:** Radiologic features of 66 HCCs and agreement between observers.

Parameters	Observer 1	Observer 2	Agreement test (kappa value)
All HCCs (66 lesions)			
*Maximum tumor diameter (mm)*	53 (17–162)	53 (15–162)	
*Signal homogeneity*			0.683
Homogenous	27 (0.41)	25 (0.38)	
Heterogeneous	39 (0.59)	41 (0.62)	
*Tumor capsule*			0.454
Completely surrounded	22 (0.33)	19 (0.29)	
Not completely surrounded	28 (0.42)	34 (0.51)	
None	16 (0.24)	13 (0.20)	
*Tumor margin*			0.513
Smooth margin	13 (0.20)	11 (0.17)	
Focal protruding	21 (0.32)	11 (0.17)	
Multiple protruding	28 (0.42)	40 (0.61)	
Fuzzy margin	4 (0.06)	4 (0.06)	
*Peritumor enhancement*			0.822
No	45 (0.68)	45 (0.68)	
Yes	21 (0.32)	21 (0.32)	
*Peritumor hypointensity*			0.782
No	37 (0.56)	40 (0.61)	
Yes	29 (0.44)	26 (0.39)	

**Table 4:** Univariate logistic regression analysis of 66 HCCs.

Parameters	Number	OR	95% CI		*p*	*R* ^2^
			LOR	UOR		
Maximum tumor diameter	66	1.024	1.008	1.041	0.003	0.144
Signal homogeneity	66	2.437	0.695	8.554	0.164	0.031
Tumor capsule	66	1.821	0.798	4.154	0.154	0.031
Tumor margin	66	4.247	1.356	13.299	0.013	0.141
Peritumor enhancement	66	7.150	2.125	24.057	0.001	0.152
Peritumor hypointensity	66	6.000	1.783	20.191	0.004	0.131
DWI	39	0.942	0.699	1.269	0.694	0.004
ADCs	39	0.915	0.140	5.995	0.927	0.000
T1 time	27	1.001	1.000	1.003	0.102	0.105
T1 ratio	27	0.121	0.000	2382.816	0.676	0.007

**Table 5:** Multiple logistic regression analysis of the 66 HCCs.

Parameters	OR	*p*	*R* ^2^	Sensitivity	Specificity
Maximum tumor diameter	*1.018*	*0.063*	*0.353*	52.9%	93.0%
Tumor margin	*3.418*	*0.102*			
Peritumor enhancement	*6.065*	*0.035*			

## Abbreviations

MVI: Microvascular invasion
HCC: Hepatocellular carcinoma
MRI: Magnetic resonance imaging
DWI: Diffusion weighted imaging
ADC: Apparent diffusion coefficients
HBP: Hepatobiliary phase
ROIs: Regions of interest
SI: Signal intensity
OR: Odds ratio
ROC curve: Receiver operating characteristic curve
AUC: Area under curve.
